# The Time Has Come to Repeal Section 43 of the Criminal Code

**DOI:** 10.1177/07067437231181831

**Published:** 2023-06-08

**Authors:** Jean-Francois Carmel, Stan Kutcher

**Affiliations:** 1 Department of Psychiatry, University of Montreal, Montreal, Quebec, Canada; 2 Department of Psychiatry, Dalhousie University, Halifax, Nova Scotia, Canada; 3 Independent Senator representing Nova Scotia in the Senate of Canada

In Canadian law, children are the only group of citizens who can be legally subjected to corporal punishment (CP). The United Nations Convention on the Rights of the Child insists on the necessity of protecting the child from all forms of physical and mental violence while in the care of the parents or legal guardians, including CP. Numerous organizations have condemned the use of CP and over 600 organizations have endorsed a Joint Statement on Physical Punishment of Children and Youth.^1^

CP has not been demonstrated to yield positive outcomes and is associated with an increased risk of depression, anxiety, oppositional behaviors, alcohol and drug use, lower school engagement, slower cognitive development, peer isolation and suicide attempts.^2,3^ To this day, one of the most contentious issues regarding CP is the question of whether there is in fact a causal relationship between CP and problematic behaviors in children. A meta-analysis conducted in 2002 found an association between parental spanking and negative outcomes.^4^ However, many criticized the methodology of the studies used for this meta-analysis. First, it was argued that since most studies were cross-sectional or retrospective, a causal relationship could not be inferred. Critics mentioned the need for more longitudinal prospective studies to avoid overreliance on retrospective recall. Second, confounding factors and selection bias represent a fundamental problem when analysing nonrandomized studies in meta-analyses. Ethical concerns have made it impossible to evaluate the impact of CP by using an experimental randomized design. However, even when reviewing experimental studies that were conducted in the 1980s and 1990s, none reported CP to be more effective than other parenting strategies.^5^ Third, it was suggested that better replications, robustness checks and falsification tests would give more statistical power to the studies. Next, it was suggested that CP should be more clearly defined and distinguished from physical abuse. Finally, it was mentioned that the negative outcomes from CP could not be generalized to all contexts, such as in combination with other punitive strategies or in other settings. Some scholars have reanalysed the results from the 2002 meta-analysis and have suggested that the effect size for the impact of CP on negative child outcomes is significantly smaller than the one for harsh physical punishment, although both are significant and positive.^6^

Following these critics, subsequent studies aimed to not only determine if there was a mere association between CP and pediatric behavioral problems, but to identify if there was a causal link between the two. Measuring CP before the onset of adverse outcomes was therefore essential in establishing causality. In order to confirm this causal relationship without experimental studies, CP must be correlated with poor outcomes, parental use of CP must precede poor outcomes and the relationship should not be accounted for by other confounding factors. An effort was made to increase the number of longitudinal prospective studies, and specific designs were used to increase the strength of the causal relationship between CP and behavioral problems. New methodological approaches and rating scales assessing parenting behaviors have been designed over the years in attempt to address some of the limitations from previous measures in recording the incidence of CP. A more specific definition of spanking was also used to clearly distinguish it from physical abuse. When controlling the direction of the associations between spanking and negative outcomes, CP was associated with lower long-term obedience, more aggressive behavior, more mental health issues, lower cognitive performance, increased risk of abuse and poorer relationship between the parent and their child.^5^

Recently, a 2016 meta-analysis reviewed 50 years of research on CP, finding that CP consistently predicted deleterious outcomes for children.^6^ This study only selected studies that used a more restrictive definition of “spanking” and more sophisticated analytical techniques, such as longitudinal studies and rigorous control of variables. It supported that CP was associated with increased aggressive and antisocial behavior even when removing studies relying on potentially abusive parenting methods. Also, while 70% of the studies were cross-sectional or retrospective, the effect sizes did not differ from the longitudinal studies. Even more recently, a 2021 meta-analysis evaluating 69 prospective longitudinal studies confirmed previous findings that CP was a predictor of increased behavioral problems, further supporting evidence of a causal relationship.^7^

There is a debate between choosing legislation either specifying limitations or a complete prohibition of CP. In Canada, the Supreme Court narrowed the application of Section 43 of the Criminal Code to “the use of minor force that is reasonable under the circumstances” and offered guidelines regarding the use of CP on children. CP should only be used by parents or caregivers, be transitory and trifling in nature, be administered between the ages of 2 and 12 years old. Furthermore, CP should not be given in retaliation for something a child did, cannot result in harm and cannot be used on a child who is incapable of learning from the situation. If physical punishment is given, the force must be minor and objects, such as belts or rulers, cannot be used. A recent study demonstrated that most cases of substantiated physical abuse in Canada would still fall within the guidelines provided by the Court.^8^ Most cases of maltreatment involve the parents, with most children being usually between 2 and 12 years old. These cases also usually do not result in physical injury and do not involve the use of an object.

Sweden was the first country to ban CP in 1979. Since then, 62 states have banned all types of CP on children. Countries prohibiting the use of CP have lower rates and faster reductions in the use of CP as well as a shift in parental attitudes towards CP.^9^ In several jurisdictions, prohibition of CP serves more as an educational rather than a punitive role, as the goal is to enhance awareness and offer assistance in preventing CP. According to many, any legal prohibition should not be punitive, but geared towards providing additional resources for parents and families in need, especially when CP is part of traditional cultural disciplinary method. Being culturally sensitive and bridging cross-cultural differences are essential in creating a legislation which takes into consideration the complex and specific needs of the community without bias, while keeping in mind children's best interests.

Beyond the debate regarding the harm caused by CP, we need to move the discussion of harm to the issue of ethical reasons for opposing CP. A false dichotomy seems to exist between CP and abuse that legitimizes physical aggression against youth. In fact, there is some irony in discipline attempts that try to reduce externalized behavior – such as aggression – by using spanking.

In sum, there is no medical or scientific reason to support CP as a child rearing tool. In Canada, children are the only group of citizens who can still be legally subjected to CP under the Criminal Code C-46, Section 43. Therefore, we see no reason why this section of the Code should remain.
